# Specific transcriptional responses induced by 8-methoxypsoralen and UVA in yeast

**DOI:** 10.1111/j.1567-1364.2007.00270.x

**Published:** 2007-07-30

**Authors:** Michèle Dardalhon, Waka Lin, Alain Nicolas, Dietrich Averbeck

**Affiliations:** 1 Institut Curie Section de Recherche, UMR2027 CNRS/I.C., INSERM, LCR n°28 CEA, Centre Universitaire d'Orsay Orsay Cedex, France; 2 Institut Curie Section de Recherche, UMR 7147 CNRS, Université Pierre et Marie Curie Paris Cedex, France

**Keywords:** DNA repair, 8-methoxypsoralen (8-MOP) plus UVA, *Saccharomyces cerevisiae*, microarrays, gene expression

## Abstract

Treatment of eukaryotic cells with 8-methoxypsoralen plus UVA irradiation (8-MOP/UVA) induces pyrimidine monoadducts and interstrand crosslinks and initiates a cascade of events leading to cytotoxic, mutagenic and carcinogenic responses. Transcriptional activation plays an important part in these responses. Our previous study in *Saccharomyces cerevisiae* showed that the repair of these lesions involves the transient formation of DNA double-strand breaks and the enhanced expression of landmark DNA damage response genes such as *RAD51*, *RNR2* and *DUN1*, as well as the Mec1/Rad53 kinase signaling cascade. We have now used DNA microarrays to examine genome-wide transcriptional changes produced after induction of 8-MOP/UVA photolesions. We found that 128 genes were strongly induced and 29 genes strongly repressed. Modifications in gene expression concern numerous biological processes. Compared to other genotoxic treatments, *c*. 42% of the response genes were specific to 8-MOP/UVA treatment. In addition to common DNA damage response genes and genes induced by environmental stresses, a large fraction of 8-MOP/UVA response genes correspond to membrane-related functions.

## Introduction

The bifunctional furocoumarin 8-methoxypsoralen (8-MOP) is a well established drug in the photochemotherapy of psoriasis and other skin diseases, including the treatment of cutaneous T-cell lymphoma (Averbeck, 1989; Zarebska *et al.*, 2000). However, psoralens such as 8-MOP also have a genotoxic potential that has been attributed to their ability to bind specifically and covalently to pyrimidine bases in DNA upon UVA irradiation (Averbeck, 1989). These compounds intercalate into DNA and undergo photocycloaddition with pyrimidines (mainly thymidine) in a sequence-specific manner to form monoadducts and interstrand crosslinks (ICLs) (Dall'Acqua *et al.*, 1970), for which 5′TpA sites are the preferred targets. ICLs induced by 8-MOP plus UVA (8-MOP/UVA) are critical genotoxic lesions and have strong antiproliferative effects (Zheng *et al.*, 2006).

The DNA lesions induced upon 8-MOP/UVA exposure differ strikingly from the pyrimidine dimers induced by UV radiation, the single- and double-strand breaks and base lesions induced by ionizing radiation and the methylated bases induced by methyl methane sulphonate (MMS). Moreover, the pyrimidine photoadditions induced by 8-MOP/UVA are clearly different from the purine mono- and diadducts induced by alkylating crosslinking agents such as nitrogen mustard and cisplatin, and they are chemically more stable (Zheng *et al.*, 2006). Due to energy transfer reactions, exposure to 8-MOP/UVA can also produce singlet oxygen, which may attack all cellular constituents (Zarebska *et al.*, 2000). Stable cyclobutane photoadducts can also be formed with unsaturated fatty acids, thus affecting membranes (Zarebska *et al.*, 2000).

With respect to the specificity and the complexity of the damage induced by 8-MOP/UVA, it was of particular interest to analyse the global transcriptional response of eukaryotic cells. We previously established genotoxicity levels and dose-response curves for this agent for the model yeast *Saccharomyces cerevisiae* (Cohen *et al.*, 2002). We showed by pulsed-field gel electrophoresis that 8-MOP/UVA treatment causes DNA double-strand breaks (DSBs) (Dardalhon & Averbeck, 1995; Dardalhon *et al.*, 1998), accompanied by the induction of DNA damage response genes such as the ribonucleotide reductase subunit gene *RNR2*, which is involved in DNA metabolism, and the DNA repair genes *RAD54* and *RAD51*, which are involved in the repair of DSBs by homologous recombination (Averbeck & Averbeck, 1994, 1998). However, the formation of these DSBs is indirect, as they result from the repair of primary ICLs (Jachymczyk *et al.*, 1981; Magaña-Schwencke *et al.*, 1982; Saffran *et al.*, 2004) through nucleotide excision repair, homologous recombination and/or postreplication repair. Nuclear excision repair and a Pso2/Msh2/Exo1-dependent pathway are both required to process ICLs in S-phase cells, prior to DSB repair (Barber *et al.*, 2005). Cell cycle arrest caused by DNA damage constitutes another response. Arrest is brought about by a network of proteins that detect damage and signal the inhibition of mitosis and the expression of damage-inducible genes through a cascade of protein kinases, including Mec1p (a homolog of the mammalian ATM/ATR checkpoint kinases), Rad53p (homologous to human Cds1p) and Chk1p (homologous to human Chk1p) (Weinert, 1998; Lowndes & Murguia, 2000). How these kinases are activated in response to DNA damage is not yet well understood, but cell cycle arrest provides an opportunity for cells to repair DNA damage before mitosis, which otherwise might lead to the segregation of incompletely replicated chromosomes or chromosomal segments that lack functional centromeres (Weinert, 1998).

In the present study, keeping in mind that transcriptional regulation does not predict the importance of a gene in resistance to a genotoxic agent (Birrell *et al.*, 2002), but helps in the characterization of the global genotoxic effect as compared to other agents, we have investigated, in a haploid yeast strain in exponential growth phase, the genome-wide transcriptional changes produced after induction of 8-MOP/UVA photolesions using complete genome ORF microarrays.

## Materials and methods

### Strains and culture conditions

The DNA repair-competent haploid strain BY4741 (MAT a, *his3*Δ, *leu2*Δ0, *met*15Δ0, *ura*3Δ) was used. Cells were precultured twice at 30°C for 12 h in liquid minimal medium [0.67% ammonium base without amino acids (Difco), 2% glucose (Merck)] supplemented with appropriate amino acids. Cell cultures in exponential phase (final concentration 10^7^ cells mL^−1^) were obtained in liquid YPD medium [0.5% yeast extract (Difco), 2% bactopeptone (Difco), 2% glucose].

### 8-MOP plus UVA (8-MOP/UVA) treatment

Cells were harvested during exponential growth phase, centrifuged, resuspended in sterile water, incubated in the presence of 5-μM 8-MOP (Sigma) in the dark for 30 min and exposed to 365-nm UVA radiation at a fluence rate of 11 J m^−2^ s^−1^ using a HPW125 Philips lamp with a Pyrex water filter. All 8-MOP/UVA experiments were performed at 20°C. Cells were irradiated with 8-MOP/UVA and resuspended in fresh YPD at the original culture volume. Culture samples were collected for fluorescence-activated cell sorting (FACS analysis), measurements of cell survival rates, and Northern blot and transcriptome analyses at different time points after photosensitizing treatment. Experiments with 8-MOP in the dark or UVA irradiation alone were conducted for different incubation times in YPD medium.

### Survival studies

Clonogenic survival of cells treated in exponential growth phase was determined as previously described by taking aliquots from treated and untreated samples (Averbeck & Averbeck, 1994). After dilution and plating on solid complete growth medium containing 2.6% agar (Difco), plates were incubated for 5 days at 30°C. Outgrowing colonies were counted and the surviving fraction was determined.

### Fluorescence activated cell sorting (FACS analysis)

Cells from exponential phase cultures were treated as previously described (Cohen *et al.*, 2002). After sonication, cells were checked by light microscopy before analysis with a FACSCalibur apparatus (Becton-Dickinson). For each experimental point, 2 × 10^4^ cells were analyzed using cellquest software.

### RNA extraction and Northern blotting

Total RNA was extracted from 0.5–1.0 × 10^8^ cells using the FastRNA-Red kit (Bio101) according to the manufacturer's instructions and subjected to Northern analysis as described (Cohen *et al.*, 2002). Blots were successively hybridized with a radio-labeled *RAD51* DNA probe (335 bp), prepared by digesting pTZ51 (a gift of F. Fabre, CEA) with ClaI and BstXI, and an *ACT1* probe (587 bp), prepared by digesting pSK-*ACT* (a gift of M. Vedel, Institut Curie) with HindIII+XbaI. Blots were scanned using a PhosphorImager system (Molecular Dynamics) and analyzed using image quant v.5.1 software (Molecular Dynamics). *RAD51* gene up-regulation was determined as previously described (Cohen *et al.*, 2002).

### mRNA preparation for microarray analysis

Total RNA was extracted using a protocol derived from the hot acid phenol extraction method as described at http://www.molecularstation.com. Total RNA (500 to 1000 μg for each sample) was used for Poly(A)+RNA isolation with the Micro-Fast Track 2.0 kit (Invitrogen) according to the manufacturer's protocol for yeast mRNA. Target samples for microarray analysis were prepared using an amino-allyl dye coupling protocol (http://www.molecularstation.com). Microarray analysis was performed with cells treated with 8-MOP/UVA, cells treated with 8-MOP in the dark without UVA (8-MOP/dark) or cells treated with UVA alone and incubated for 3 h or various times (1–5 h) in complete growth medium. Aliquots from each time point were used to generate cDNA probes labeled with Cy5-dUTP or Cy3-dUTP, and differentially labeled probes were combined and hybridized to yeast genomic microarrays.

### Experimental design

The experimental scheme consisted of incubating cells with 5 μM 8-MOP in the dark for 30 min. Part of this material was immediately frozen and served afterwards for RNA extraction and cDNA labeling (control 8-MOP exposure), and the remainder was halved. For 8-MOP/dark treatments, one fraction was incubated in complete growth medium for 0–5 h at 30°C. For 8-MOP/UVA treatments, the other fraction was exposed to UVA (5 kJ m^−2^) and incubated in complete growth medium for 0–5 h at 30°C. Samples were withdrawn hourly, frozen and used for generating labeled cDNA probes. For each time point, labeled cDNA prepared from 8-MOP/dark- or 8-MOP/UVA-treated cells was hybridized with control labeled cDNA to yeast genomic microarrays. To strengthen the kinetic results, we performed additional experiments with labeled cDNA from 8-MOP/dark-treated cells and labeled cDNA from 8-MOP/UVA- or 8-MOP/dark-treated cells that were incubated for 3 h in complete growth medium. Although previous studies demonstrated that UVA radiation alone at this dose level, as well as 8-MOP/dark treatment alone, has no significant effect on survival, mutation or gene induction (Averbeck & Averbeck, 1998; Cohen *et al.*, 2002), we also hybridized labeled cDNA prepared from untreated samples to microarrays together with labeled cDNA prepared from samples treated with UVA alone (5 kJ m^−2^) and incubated for 3 h in complete medium. In spite of the fact that changes by a less than a factor of 2 can be significant, and in order to identify only those transcripts that were specifically induced by 8-MOP/UVA treatment, genes were defined as highly differentially regulated if they exhibited an at least twofold change in the level of expression, and genes affected by exposure to UVA alone or to 8-MOP alone after incubation in growth medium were discarded. The intra-experimental variations were correct and the levels of *RAD51* mRNA in each experiment were in accord with Northern measures. The mean value from three experiments with cells incubated for 3 h after 8-MOP/UVA- and 8-MOP/dark treatment is presented, as well as with cells incubated for 3 h after UVA-alone treatment.

### Microarray hybridization, data acquisition and analysis

Yeast coding regions were amplified by PCR using the complete set of Yeast GenePairs Primers purchased from Research Genetics. After isopropanol precipitation, PCR products were resuspended in 3X SSC. DNA was printed on polylysine-coated glass slides or Corning Ultra Gaps II slides using a Microgrid TAS arraying robot (Biorobotics). The slides were processed, hybridized at 63°C, and washed as described (http://www.molecularstation.com). For microarray analyses, slides were scanned using a Genepix 4000A scanner (Axon Instruments) and images were processed using genepix pro 4.0 software. Data from poor quality spots and spots with a total intensity below 500 were eliminated. The local background median intensity was subtracted from the median spot intensity before calculating the intensity ratio (Cy5 median intensity/Cy3 median intensity) for each spot. Data storage, filtering, global normalization and signal intensity ratio calculation were performed using the base (BioArray Software Environment) 1.0.7 program. Genes were annotated using Gene Ontology terms (Ashburner *et al.*, 2000) provided at the Saccharomyces Genome Database website (SGD) (Cherry *et al.*, 1998) (GO Consortium: http://www.geneontology.org; SGD: http://www.yeastgenome.org). Gene regulatory information was obtained using the Yeast Proteome Database (YPD) (Costanzo *et al.*, 2000). Genes for which the log_2_ Cy5/Cy3 ratio was >1 or <−1 (2 and 0.5-fold differences in expression, respectively) were defined as differentially regulated.

### Online supplementary material

As previously described (Sollier *et al.*, 2004), microarray details are provided at http://www.ebi.ac.uk/aerep/(query for arrays, accession number a-mexp-40). Probe details, microarray hybridization protocols and data are available at http://microarrays.curie.fr/. A list of genes with ≥2-fold altered levels of expression after 8-MOP/UVA treatment is presented in supplementary Table S1 and as a function of posttreatment incubation time in supplementary Table S2. Lists of 8-MOP/UVA response genes encoding proteins with membrane related functions and comparison with other genotoxic responses in yeast are presented in supplementary Tables S3 and S4, respectively. A Treeview analysis (Eisen *et al.*, 1998) of 8-MOP/UVA response genes whose expression overlaps with that induced by histone depletion (Wyrick *et al.*, 1999) is shown in supplementary Fig. S1.

## Results and discussion

### Cell cycle analysis and *RAD51* mRNA expression after 8-MOP/UVA treatment of haploid yeast cells

**Fig. 1:**
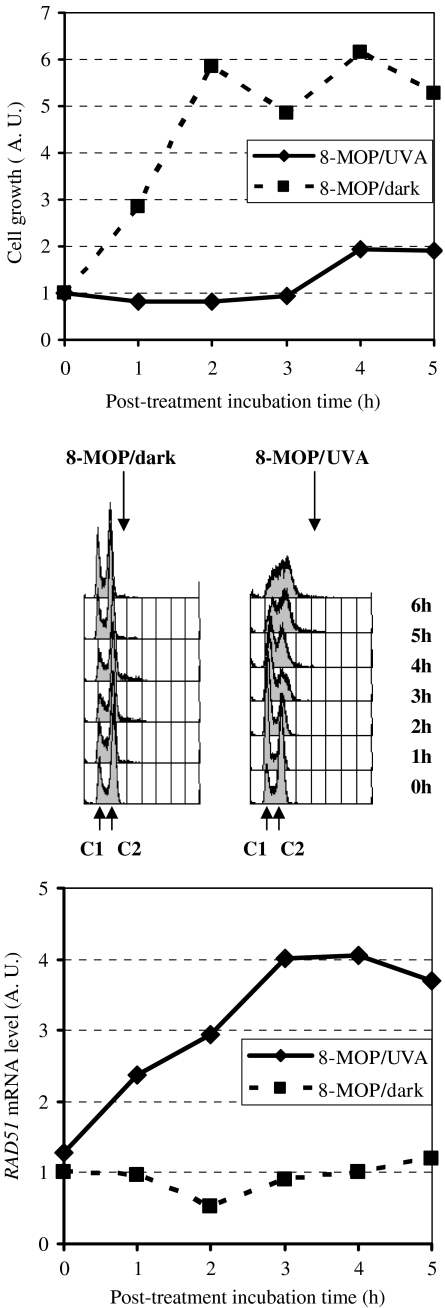
Responses to 8-MOP damage in wild-type cells following treatment with 5 μM 8-MOP plus 5 kJ m^−2^ UVA as compared to 8-MOP/dark-treated cells (exposure to 5 μM 8-MOP in the dark). Top: growth of 8-MOP/UVA- and 8-MOP/dark-treated cells after posttreatment incubation in complete growth medium. Middle: cell cycle profiles of 8-MOP/UVA- and 8-MOP/dark-treated cells during posttreatment incubation in complete medium as monitored by FACS analysis. C1 denotes a single genome content (equivalent to that of G1 haploids) and C2 denotes a double genome content (equivalent to that of G2 haploids). Bottom: Northern blot analysis of *RAD51* mRNA levels in 8-MOP/UVA- and 8-MOP/dark-treated cells. Induction was normalized to actin (*ACT1*) transcript levels and is given in arbitrary units.

To study transcriptome modifications after treatment with 8-MOP/UVA, we chose experimental conditions that allowed *c*. 20% survival of haploid wild-type cells, incubation in 8-MOP (5 μM) followed by exposure to 5 kJ m^−2^ UVA. 8-MOP/UVA-treated cells did not resume growth until *c*. 3 h posttreatment, whereas 8-MOP/dark-treated cells continued to grow (Fig. 1). FACS analysis showed that the proportions of 8-MOP/dark-treated cells in G1, S, and G2/M remained constant during the incubation period (Fig. 1). However, 8-MOP/UVA-treated cells showed a modified cell cycle distribution during posttreatment incubation (Fig. 1). Finally, *RAD51* mRNA transcript levels increased immediately after 8-MOP/UVA exposure, reaching a maximum between 3 and 4 h posttreatment with an approximately fourfold difference in the expression level, whereas the level of the *RAD51* transcript remained stable in 8-MOP/dark experiments (Fig. 1). Microarray analysis was thus performed under these well-characterized conditions. (Averbeck & Averbeck, 1998; Cohen *et al.*, 2002)

### Global changes in gene expression

Genes that responded similarly to 8-MOP/UVA treatment over the time course and after 3 h incubation were designated as response genes. Among the 6217 ORFs spotted on our microarrays, 157 genes exhibited a reproducible modification in expression upon 8-MOP/UVA treatment (listed in supplementary Table S1). Of these, 128 ORFs showed a significant increase in expression. The greatest extent of induction was 16-fold for *RNR3*, which encodes a large subunit of ribonucleotide-diphosphate reductase, and for *NCA3*, which encodes a member of the SUN family involved in processes such as DNA replication, ageing and mitochondrial biogenesis. Twenty-nine genes were down-regulated. The strongest repression was 3.8-fold for the ORF *YGR273c* of unknown function.

The analysis of the kinetics of gene induction following genotoxic damage was informative about the timing of events during the posttreatment period. Indeed, 41 genes were induced rapidly within the first hour, whereas others were induced 2 or 3 h later (supplementary Table S2). Genes already known to be involved in DNA metabolism (*RNR2*, *RNR3*, *RNR4*) or in DNA repair (*RAD51*, *DUN1*, *DIN7*) were induced after 1 or 2 h of posttreatment incubation time, respectively. Genes such as *SLT2*, *ECM3*, *GUP2* and *PDE2*, involved in signal transduction, belong to the early genotoxic response. The high levels of expression of those genes during the first hour were not maintained throughout the entire posttreatment period but rapidly declined after reaching peak levels (data not shown). Analysis of the loci of responsive ORFs according to SGD annotations (Cherry *et al.*, 1998) shows that they are distributed throughout the genome (data not shown).

### Cellular localization of products of 8-MOP/UVA response genes

We analysed the cellular localization of proteins encoded by genes showing altered expression upon 8-MOP/UVA treatment, in accordance with previous determinations made by O'Shea with GFP-tagged proteins (Huh *et al.*, 2003) (data not shown). This analysis showed that all cellular compartments, including the nucleus, are represented. Gene products located in the cytoplasm or in membranes are prominent, indicating that cellular constituents other than those involved in DNA metabolism are affected by exposure to 8-MOP/UVA. We found a higher-than-expected involvement of genes encoding membrane-bound proteins: 31.3%, vs. 23% for the genome as a whole.

### Categories of gene function affected by 8-MOP/UVA treatment

**Fig. 2:**
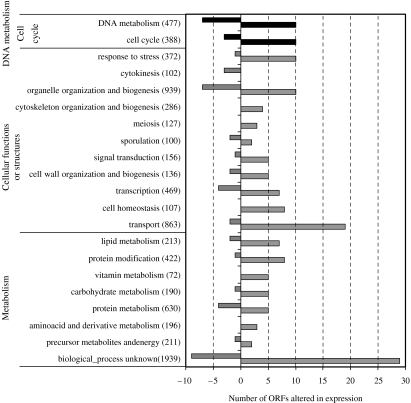
Assignments of 8-MOP/UVA response genes to biological processes (up-regulated genes are indicated by positive numbers; down-regulated genes are indicated by negative numbers). The genome-wide number of ORFs in each process is given in brackets. Categories are derived from the SGD database. Some ORFs fall into multiple categories.

**Fig. 3:**
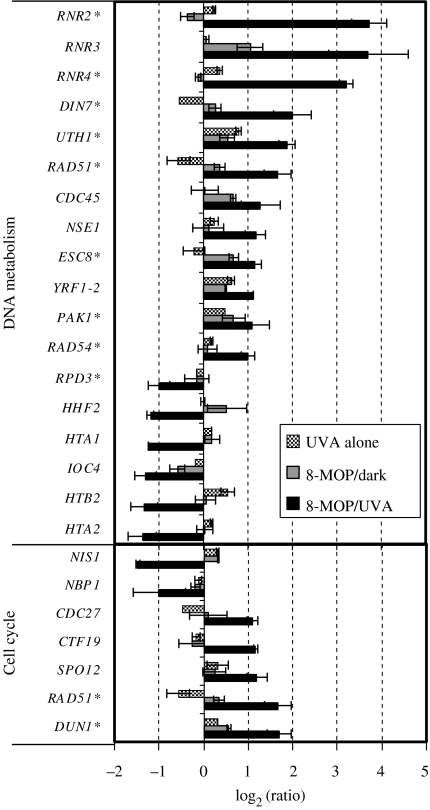
Specific genes involved in DNA metabolism and cell cycle are activated or repressed in cells treated with 8-MOP/UVA. Plotted data are the mean log_2_ ratios. (^*^Genes that fall into multiple categories.)

**Fig. 4:**
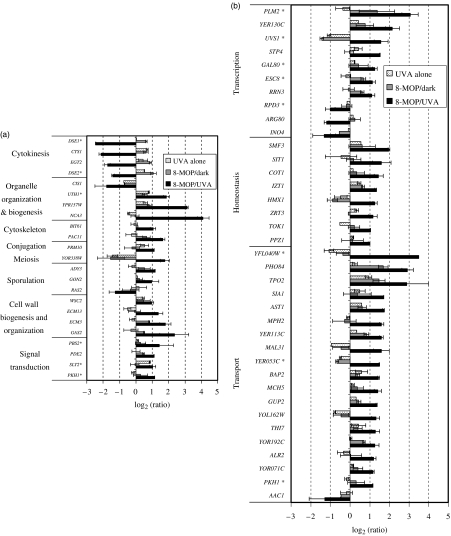
Specific genes involved in cellular functions or structures are activated or repressed in cells treated with 8-MOP/UVA. Plotted data are the mean log_2_ ratios. (a) Genes implicated in, cytokinesis, organelle, cytoskeleton, conjugation, meiosis, sporulation, cell wall, and signal transduction. (b) Genes implicated in transcription, homeostasis, and transport. (^*^Genes that fall into multiple categories.)

**Fig. 5:**
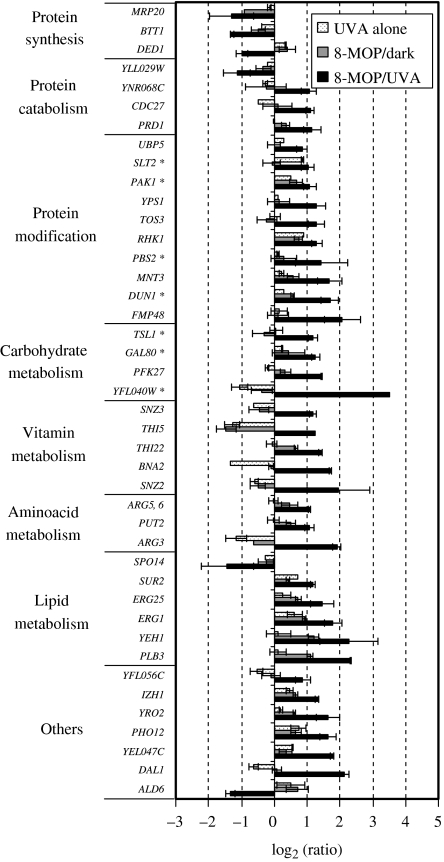
Specific genes involved in cell metabolism are up-regulated or down-regulated in cells treated with 8-MOP/UVA. Plotted data are the mean log_2_ ratios. (*Genes that fall into multiple categories.)

To gain an overview of the gene functions modified after 8-MOP/UVA, we assigned them to functional categories as defined by the Gene Ontology (GO) project (Fig. 2). These assignments show that 8-MOP/UVA response genes are implicated in a wide variety of biological processes that concern three main activities: (1) DNA metabolism and cell cycle-related functions; (2) cellular functions and maintenance of cellular structures; and (3) general metabolism. 8-MOP/UVA response genes involved in DNA metabolism and cell cycle control, in cellular functions or structures and in metabolism are presented and quantified in Figs 3, 4 and 5, respectively.

### Response genes involved in DNA damage repair and the cell cycle

Genes known to be up-regulated in response to DNA damage induced by MMS and ionizing radiation (Jelinsky *et al.*, 2000), such as *RAD51, RAD54*, *RNR2*, *RNR4*, *DIN7* and *RNR3*, were also up-regulated after 8-MOP/UVA exposure (Fig. 3). The important role of recombinational pathways in interstrand DNA cross-link repair (Saffran *et al.*, 2004) is consistent with the induction of the recombinational repair genes *RAD54* and *RAD51* we observed. However, we found that genes involved in nucleotide excision repair (*RAD2*, *RAD16* and *RAD23*) and postreplication repair (*RAD18*) taking part in repair of 8-MOP/UVA DNA lesions (Saffran *et al.*, 2004) were unaffected.

Other notable genes relevant to DNA repair were induced, including *NSE1*, which encodes an essential nuclear protein that has a nonstructural role in the maintenance of chromosomes and which is a component of the *SMC5-SMC6* complex involved in DNA repair, and *UTH1*, a member of the SUN family involved in mitochondrial autophagy, the oxidative stress response, the starvation response, mitochondrial biogenesis, and cell death. *PAK1*, which encodes an upstream kinase for the *SNF1* complex, was also present. Moreover, we identified *YRF1-2*, a helicase that is highly expressed in mutants lacking the telomerase component *TLC1*. Other prominent genes were *CDC45* and *ESC8*, which encode a DNA replication initiation factor and a protein involved in telomeric and mating-type locus silencing, respectively.

Among the repressed genes, *RPD3* encodes a histone deacetylase, and *IOC4* is implicated in chromatin remodeling. Furthermore, we observed repression of the histone genes *HTA1*, *HTA2* and *HTB2* (Fig. 3). Also, the induction of *YRF1-2* as well as other subtelomeric genes such as *YFL061W*, *YFL065C* and *YFL066C* (supplementary Table S1) may result in structural modifications of chromatin relevant to DNA repair. These proteins share near identity to other subtelomerically-encoded proteins that are members of the Y' expression cluster (Yamada *et al.*, 1998).

Several genes involved in the cell cycle were up-regulated after 8-MOP/UVA treatment (Fig. 3). These include *DUN1*, which encodes a kinase required for the DNA damage-induced transcription of certain target genes and the control of the DNA damage response, *SPO12*, a positive regulator of the exit from mitosis, *CTF19*, an outer kinetochore protein that is required for accurate mitotic chromosome segregation, and *CDC27*, a subunit of the anaphase-promoting complex. Among repressed genes we found *NBP1*, which encodes a component of the mitotic apparatus, essential for the G2/M transition, and *NIS1*, which encodes a protein localized in the bud neck at the G2/M transition that may be involved in a mitotic signaling network.

### Response genes involved in cellular structures and functions

Several genes involved in cytokinesis, in organelle organization and biogenesis, in the cytoskeleton structure as well as genes involved in conjugation, meiosis, spore wall formation and sporulation were modified after 8-MOP/UVA treatment suggesting that cellular integrity must be restored before cell division (Fig. 4a). Another important cellular compartment affected by 8-MOP/UVA treatment is the mitochondrion, which functions in energy metabolism and apoptosis.

8-MOP/UVA response genes whose products localize to mitochondria include *DIN7*, *YEL047C*, *COT1*, *FMP48*, *PIC2*, *FMP50*, *YRO2*, *PUT2*, *ARG5, 6*, *FMP52* and *MRP20*. Also, genes involved in mitochondrial organization and biogenesis were strongly induced, including *NCA3*, *UTH1* and *YPR157W* (Fig. 4a). These 8-MOP/UVA mitochondrial effects are in accord with previous work describing the induction of respiratory deficiency in yeast (Averbeck, 1989), mitochondrial dysfunction following the opening of permeability transition pores (Canton *et al.*, 2002), and the induction of apoptosis after psoralen plus UVA treatment (Viola *et al.*, 2004).

Cell wall maintenance genes such as *WSC2*, *ECM13*, *ECM3* and *GAS2* were induced, indicating that 8-MOP/UVA treatment affects cell wall components that must be reconstructed (Fig. 4a). Genes implicated in cellular signaling were upregulated as well (Fig. 4a). These include *SLT2* and *PKH1* (encoding a serine/threonine MAP kinase), *PBS2* (protein tyrosine kinase) and *PDE2* (cAMP-specific phosphodiesterase). Interestingly, *SLT2* is slightly up-regulated after UVA alone. The activation of MAP kinases by 8-MOP/UVA is in line with previous work. For example, in human cells, cyclobutane adducts of fatty acids formed by 8-MOP/UVA can be released from modified phospholipids through phospholipase A2 hydrolysis, and these adducts can act like diacylglycerol, a second messenger responsible for various biological effects, including melanogenesis (Zarebska *et al.*, 2000). It has been proposed that 8-MOP-fatty acid adducts activate protein kinase C to enhance UVA-induced melanogenesis (Zarebska *et al.*, 2000), pointing to the importance of psoralens and UVA-induced photodamage of membranes.

Expression of several transcription factors was affected by 8-MOP/UVA treatment (Fig. 4b). However, by examining the Biomolecular Interaction Network Database (BIND) provided at SGD and gene regulatory information provided at YPD, we identified only a few 8-MOP/UVA response genes among their known or inferred targets. This finding suggests that the altered expression of these transcription factors does not account for the global 8-MOP/UVA transcriptional response. 8-MOP/UVA-induced genes are involved in transport (Fig. 4b). These include up-regulated genes encoding proteins with diverse transporter activities for amino acids, polyamine, hexoses, maltose, glycerol, thiamine, phosphate, protons and cations. Among homeostasis genes, several regulatory ion flux genes (involved in iron, zinc, sodium or potassium metabolism) were induced by 8-MOP/UVA (Fig. 4b). The induction or repression of transport-related genes may be necessary to maintain cellular homeostasis when membranes are damaged.

Analyses of our results reveal that membranes are important targets for 8-MOP/UVA because we find an enrichment concerning gene products located in the membranes as compared to the genome as a whole (see above). Genes encoding proteins with membrane-related functions (transport, homeostasis, signal transduction, cell wall maintenance) are numerous. The expression of several genes encoding proteins with transmembrane segments, such as *YR02*, *YHL044W*, *RTA1*, *YCR061W*, *GUP2*, *YER113C*, *WSC2*, *YOR071C*, *TPO2* and *PRM10* are modified after 8-MOP/UVA treatment. In addition, among the genes with expression specifically modified by 8-MOP/UVA, several are also induced by the membrane-perturbing agent chitosan (Zakrzewska *et al.*, 2005) and/or by transient cell wall damage (Garcia *et al.*, 2004) (supplementary Table S3). Altogether, these results suggest that 8-MOP/UVA lesions at membrane level play an important role on variations in the transcript levels. The relatively high proportion of genes with membrane-related functions in 8-MOP/UVA response genes might be explained by the fact that cellular targets affected by psoralen plus UVA treatment include not only DNA, with the induction of mono- and diadducts (Averbeck, 1989), but also cytoplasm and cell membranes, with the oxidation of phospholipids, and membrane proteins (Zarebska *et al.*, 2000). Interestingly, some 8-MOP/UVA-sensitive mutants show specific alterations of membrane lipids (Querol *et al.*, 1994; Brendel *et al.*, 2003). Indeed, oxygen-dependent photoreactions between furocoumarins and cell membrane constituents can lead to lipid peroxidation and protein cross-linking, and oxygen-independent photoreactions can lead to C4 cyclo-addition between furocoumarin and unsaturated fatty acids, thereby modulating membrane functions and intracellular pathways (Dall'Acqua & Martelli, 1991; Zarebska *et al.*, 2000). The combined treatment 8-MOP/UVA leads to significant production of reactive oxygen species (ROS). This might be one possible explanation for the results that differential expression of significantly more genes coding for various cellular structures and functions (like membranes, cytoplasmic vesicles, transport) is found. It would be informative to use ROS scavengers before expression analysis or to compare 8-MOP/UVA effects with typical ROS-producing agents.

### Response genes implicated in carbohydrate, amino acid and lipid metabolism

The lesions induced in DNA and in other macromolecules by 8-MOP/UVA treatment led to an altered metabolic program, with the induction of genes involved in carbohydrate or vitamin metabolism (Fig. 5). Moreover, genes involved in protein degradation were modified, suggesting that proteins altered by 8-MOP/UVA treatment may be targeted for removal and that the specific elimination of certain proteins may be important for cellular recovery. Indeed, evidence for new protein synthesis is suggested by the increased expression of genes involved in amino acid metabolism. Interestingly, several proteins involved in posttranslational modification were up-regulated, indicating that cellular responses to DNA damage often entail processes such as phosphorylation and proteolytic cleavage that modify protein–protein and protein–DNA interactions for efficient repair.

Genes involved in lipid metabolism were also significantly induced. Modifications in the expression of genes associated with cellular metabolism were also observed after treatment with other genotoxic agents (Gasch *et al.*, 2001). The link between modifications in gene expression and the global cellular response to 8-MOP/UVA treatment (Fig. 5) may reflect the requirement for the repair or *de novo* synthesis of damaged biomolecules, including lipids and proteins as well as DNA.

### Comparison of the transcriptomes of cells treated with 8-MOP/UVA and other genome-wide studies as well as the transcriptomes of cells treated with other genotoxic, cytotoxic or stress-inducing agents highlights the specificity of the 8-MOP/UVA response

Comparison of our data with other transcriptome studies demonstrates that 10–20% of the genes affected by 8-MOP/UVA treatment also show modified expression over the cell cycle (Spellman *et al.*, 1998), during the diauxic shift (DeRisi *et al.*, 1997), in response to alpha factor (Hughes *et al.*, 2000) or in the presence of glycosylation defects (Cullen *et al.*, 2004). However, there was little overlap with PHO pathway-regulated genes (Ogawa *et al.*, 2000). This expression profile suggests that in addition to the DNA damage response, a global cellular response is mounted after 8-MOP/UVA treatment.

**Fig. 6:**
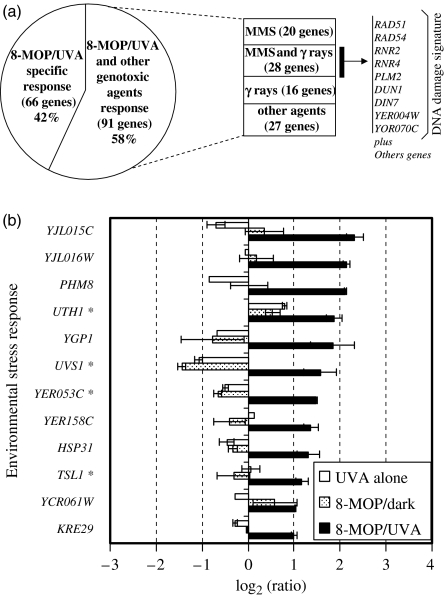
(a) Number and percentage of genes modified by 8-MOP/UVA that are specific to this treatment or that are also induced by other genotoxic agents (details in supplementary Table S4). (b) Genes encoding components responding to environmental stress are up- or down-regulated after treatment of cells with 8-MOP/UVA. Plotted data are the mean log_2_ ratios. (^*^Genes that fall into multiple categories.)

The genes that were up- or down-regulated after 8-MOP/UVA exposure (see supplementary Table 1) were compared to those affected by other genotoxic agents (Jelinsky & Samson, 1999; Jelinsky *et al.*, 2000; Gasch *et al.*, 2001; Keller-Seitz *et al.*, 2004; Mercier *et al.*, 2004, 2005; Caba *et al.*, 2005). Among the 8-MOP/UVA-induced genes, 58% are also induced by ionizing radiation, MMS or other agents, suggesting that the 42% remaining induced transcripts are specific for 8-MOP/UVA treatment (Fig. 6a and supplementary Table S4). However, it cannot be excluded that those transcripts could also be activated by other types of DNA lesions. Among the genes induced by genotoxic agents, we found nine (Fig. 6a) belonging to the DNA damage signature defined by Gasch *et al.* (2001). In a previous study, Workman *et al.* (2006) constructed a global model of transcriptional networks activated by MMS, and some of the genes in this network were also induced (*RNR2*, *RNR4*, *DIN7*, *DUN1*, *ARG3*, *AAD6*, *YLR297W* and *YGR146C*) or repressed (*ARG80*, *INO4* and *EGT2*) by 8-MOP/UVA. Some 8-MOP/UVA-modified genes are also influenced by various environmental stresses (Gasch *et al.*, 2000). The genes listed in Fig. 6b also respond to heat shock (*HSP31* and *TSL1*), osmotic shock (*YGP1*), oxidative stress (*UTH1*), and nutrient limitation (*YGP1*) (Gasch *et al.*, 2000; Causton *et al.*, 2001; Gasch & Werner-Washburne, 2002). According to the GO process ‘stress’, we found that in addition to the genes presented in Fig. 6b, the 8-MOP/UVA-modified genes *DIN7*, *DUN1*, *NSE1*, *RAD51*, *WSC2*, *HTA2*, *HTA1* and *RPD3* are also involved in stress responses. Thus, in addition to genotoxic and specific responses, a general stress response (13% of all modified genes) is mounted in *S. cerevisiae* cells after 8-MOP/UVA treatment.

Interestingly, a comparison of the total number of genes affected by 8-MOP/UVA alone to that affected by 8-MOP/UVA and other genotoxic agents shows that they differ with respect to intracellular localization, as defined by their GO profiles (data not shown). Gene products specifically modified by 8-MOP/UVA are in all compartments but gene products that localize to the cytoplasm, to membranes and to the nucleus are prominent. These biased localization patterns emphasize that the cellular targets affected by psoralen plus UVA treatment include not only DNA, but also proteins and lipids, with deleterious consequences for the cytoplasm and cellular membranes. Genes specifically modified by 8-MOP/UVA are likely critical for counteracting these effects as well as for ensuring the repair of DNA lesions induced by this agent.

### Involvement of chromatin-modifying activities in gene expression responses after 8-MOP/UVA treatment

To determine whether 8-MOP/UVA response genes are affected by chromatin dynamics, we compared our data with those obtained by analysis of deletion mutants or by employing experimental conditions that interfere with chromatin structure and function (Wyrick *et al.*, 1999; Sudarsanam *et al.*, 2000; Meneghini *et al.*, 2003; Kobor *et al.*, 2004; Mizuguchi *et al.*, 2004; Morrison *et al.*, 2004; van Attikum *et al.*, 2004). Twenty-seven percent of 8-MOP/UVA-modified genes were also modified in *snf2* and/or *swi1* deletion mutants (Sudarsanam *et al.*, 2000), indicating that the *S. cerevisiae* Snf/Swi complex, which controls transcription and the chromatin structure of particular genes *in vivo* and remodels nucleosomes *in vitro*, is implicated in gene expression following 8-MOP/UVA exposure. A few genes affected by 8-MOP/UVA (about 15%) are also modified in *swr1*Δ cells, in *htz1*Δ cells and in *sir2*Δ cells (Meneghini *et al.*, 2003; Kobor *et al.*, 2004; Mizuguchi *et al.*, 2004). This finding also suggests that under our experimental conditions, the replacement of histone H2A by H2.Z in nucleosomes as well as a Sir contribution to telomeric silencing may influence gene expression after 8-MOP/UVA treatment. In addition, there was a small overlap with the transcriptomes of *ssn6* and *tup1* mutants, which are impaired in transcription (Hughes *et al.*, 2000). Interestingly, only a few 8-MOP/UVA response genes (5%) are also affected in *ino80* cells (van Attikum *et al.*, 2004). The evolutionarily conserved *INO80* chromatin remodelling complex directly participates in the repair of DSBs in yeast (Morrison *et al.*, 2004). Based on transcriptional analysis, *INO80* does not appear to regulate homologous recombination at the transcriptional level but it may influence nonhomologous end joining, which has a modest role in the repair of the photoadducts and DSBs that indirectly result from 8-MOP/UVA exposure (Cohen *et al.*, 2002).

The most striking correlation observed was that between the transcriptional profiles caused by 8-MOP/UVA treatment and by histone H4 depletion (Wyrick *et al.*, 1999). Approximately 62% (97/157) of 8-MOP/UVA response genes are also affected by H4 depletion (supplementary Fig. S1). Although the reduction of nucleosome content caused by H4 depletion affects many genes (*c*. 25%) (Wyrick *et al.*, 1999), this overlap of 62% is highly significant (χ^2^=24.9, *P*<0.001) and, interestingly, largely covers the entire spectrum of biological functions ranging from DNA damage response genes to membrane-specific genes. This correlation may reflect changes in chromatin structure that are likely required to process the ICLs induced by 8-MOP/UVA, which ultimately leads to DSB formation (Dardalhon *et al.*, 1998).

### A specific 8-MOP/UVA transcriptional profile

The damage response to the genotoxic agent 8-MOP/UVA includes expression of genes involved in DNA maintenance and repair. However, our results also reveal that membranes are important targets for 8-MOP/UVA as we find an enrichment concerning the membranes as compared to the genome as a whole. Modifications of membrane lipids and proteins and of mitochondrial functions are closely linked to the effective processing of 8-MOP/UVA-induced DNA photolesions by these repair systems. In addition, the unexpected connection between H4 depletion and the transcriptional effects caused by 8-MOP/UVA is very interesting and warrants further studies.

Interestingly, mutants deleted for some 8-MOP/UVA-inducible genes are sensitive to genotoxic agents. For example, according to YPD gene information, mutants of *NSE1*, *ESC8*, *EGT2*, *SPO12*, *YCR146C*, *YML002W*, *YMR144W*, *YLL029W* and *YPS1* are sensitive to MMS, mutant of *YNR068C* is sensitive to gamma radiation, and mutants of *YCR061W*, *STP4* and *YHL044W* are sensitive to cisplatin. As described by Birrell *et al.* (2002) transcriptional regulation does not necessarily predict the importance of a gene in resistance to a genotoxic agent. To further define the relationship between induced genes and genotoxic consequences after 8-MOP/UVA treatment, the survival responses of mutants deleted for genes that are specifically induced by 8-MOP/UVA will be of particular interest.

Our elaboration of the 8-MOP/UVA transcriptional response is the first step in identifying the mechanisms involved in the effectiveness and genotoxic consequences of photochemotherapeutic 8-MOP/UVA treatments. However, the use of a haploid strain may not fully represent the ploidy-specific response of human cells. Therefore, further work using a homologous diploid yeast strain will be of interest.

## Statement

Re-use of this article is permitted in accordance with the Creative Commons Deed, Attribution 2.5, which does not permit commercial exploitation.

## Supplementary Materials

The following supplementary material is available for this article:

Table S1 List of genes with altered expression after 8-MOP/UVA treatment.

Table S2 Genes with altered expression after 8-MOP/UVA treatment as a function of post-treatment incubation time.

Table S3 8-MOP/UVA response genes encoding proteins with membrane related functions.

Table S4 Comparison with other genotoxic responses in yeast.

Fig. S1 8-MOP/UVA response genes that overlap with genes modified by H4 depletion as described by Wyrick *et al.*, 1999.

This material is available as part of the online article from: http://www.blackwell-synergy.com/10.1111/j.1567-1364. 2007.00270.x (This link will take you to the article abstract).
