# Supplementary material for: Specific transcriptional responses induced by 8-methoxypsoralen and UVA in yeast
Source: FEMS Yeast Res. 2007 Jul 30;7(6):866–78. doi: 10.1111/j.1567-1364.2007.00270.x (PMC2040189; doi:10.1111/j.1567-1364.2007.00270.x)
Supplement: Fig. S1 — 8-MOP/UVA response genes that overlap with genes modified by H4 depletion as described by Wyrick et al., 1999. [file fyr0007-0866-s5.ppt]

## Slide 1
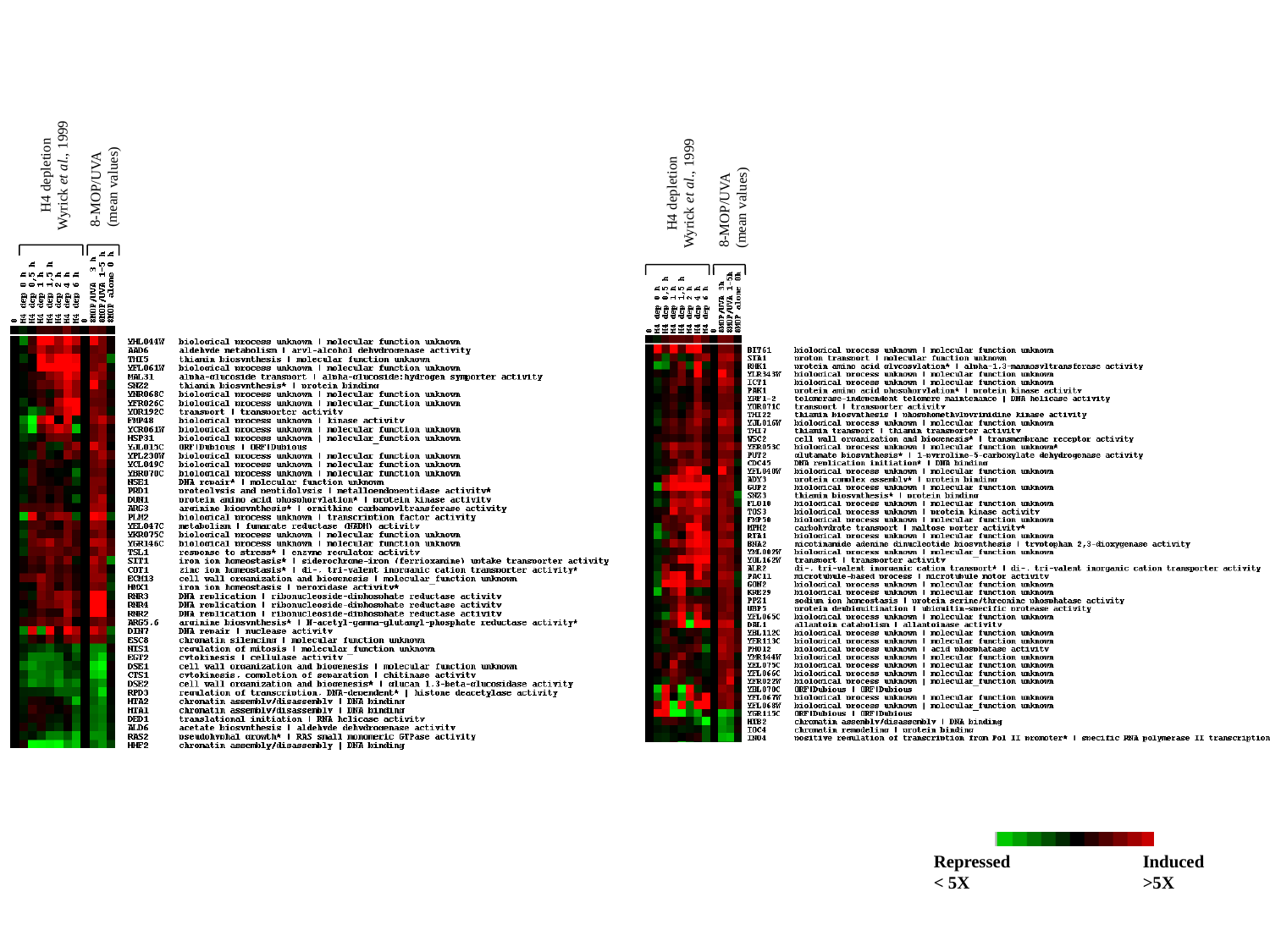

H4 depletion
Wyrick et al., 1999
8-MOP/UVA
(mean values)
H4 depletion
Wyrick et al., 1999
8-MOP/UVA
(mean values)
Repressed
< 5X
Induced
>5X
